# Study on Bending Performance of High-Ductility Composite Slab Floor with Composite Ribs

**DOI:** 10.3390/ma18030658

**Published:** 2025-02-02

**Authors:** Yuchen Jiang, Libo Liu, Xiaolei Wang, Run Liu, Haibo Yang

**Affiliations:** 1 College of Civil Engineering, Hebei University of Engineering, Handan 056038, China; jyc13361658805@163.com (Y.J.); wangsanshi2003@163.com (X.W.); 2 Institute of Geotechnical Engineering, Tianjin University, Tianjin 300072, China; liurun@tju.edu.cn; 3 College of Materials Science and Engineering, Harbin Institute of Technology, Habin 150001, China; yanghb@hit.edu.cn

**Keywords:** composite slab floor, high-ductility concrete, bending performance, free support, flange plate

## Abstract

In order to solve the problems of high production cost and complex control of the inverted arch of an unsupported prestressed concrete composite slab, a flange truss high-ductility concrete composite slab floor is proposed to change the structure and pouring material to meet the requirements of no support during construction. The crack distribution and bending performance of the flange truss high-ductile concrete composite slab floor (CRHDCS) under different structures are clarified through the test and numerical analysis of four different rib plate structure floors. According to the analysis results, the calculation formulas of the cracking moment and short-term stiffness before cracking are modified, and the equivalent short-term stiffness formula of a single web member of the “V” truss to this kind of bottom plate is established. The results show that, unlike the short-term stiffness-change law of typical concrete composite slabs after cracking, the short-term stiffness of the designed bottom plate in this paper includes a short-term increase stage. The numerical simulation results are the same as the experimental ones; the maximum error is 10%. The maximum errors between the modified cracking moment and the short-term stiffness calculation formula are 6% and 8%, respectively. The influence rates of removing flange plate, truss-inverted binding, and adding rib plate on the cracking bending moment of foundation structure are −81.5%, 11.0%, and 22.2% respectively, and the influence rates on short-term stiffness are −87.6%, −1.5%, and 37.5% respectively.

## 1. Introduction

**Figure 1:**
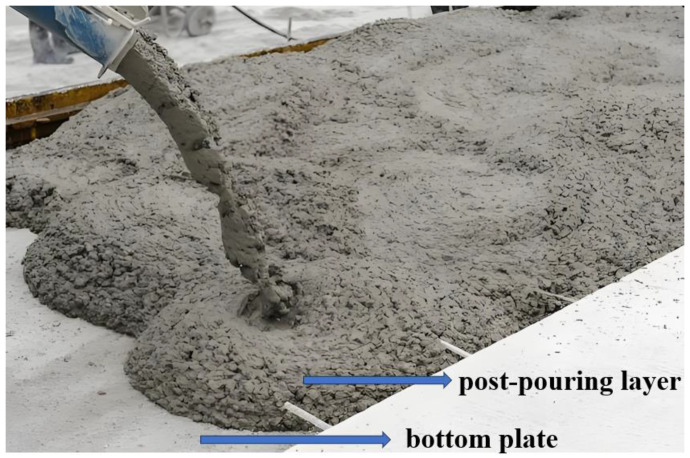
Composite slab foundation structure diagram.

The composite slab comprises a prefabricated bottom plate and a post-pouring layer [1]; the composite slab foundation structure diagram is shown in Figure 1. Since its bottom plate is a prefabricated component and can be used as a post-pouring layer template, the construction technology of the composite plate is simple [2]. Thus, it has the advantages of reducing the construction period, cost, and environmental protection, which is widely used in prefabricated buildings [3]. The composite slab floor is divided into horizontal and vertical applications. The building uses the two floors and the post-pouring layer as floor and wall components [4]. The traditional composite slab floor has some problems in horizontal application, such as challenging pipeline laying and high support costs. A large-span floor cannot meet the construction safety requirements [5].

**Figure 2:**
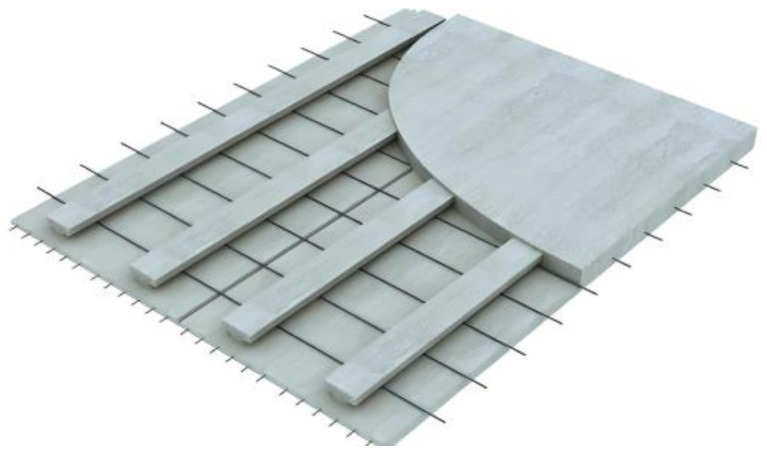
Concrete rib composite slab floor.

Due to the above problems, the main research direction of the composite plate–bottom plate is how to design the bottom plate so that the composite plate not only guarantees the design safety requirements but also makes the construction convenient and reduces costs [6]. A large number of scholars have proposed a new type of composite slab floor by changing the structure of the floor and the way of pouring materials. The specific research results are shown in the following [7]. The transformation of the floor structure is mainly the transformation of the rib form. At present, a large number of rib forms are proposed, including truss ribs, steel pipe truss ribs, steel ribs, inverted “T”-type concrete ribs, and slotted concrete ribs [6,8,9,10,11,12]. The concrete rib composite slab floor is shown in Figure 2. All kinds of ribs effectively improved the bending performance of the precast floor and the composite slab. Still, the rib structures, such as steel ribs and ordinary concrete ribs, increased significantly the production cost of the floor. The production cost of truss ribs is low, and the problem of pipeline laying is effectively solved [13,14,15]. However, only the truss is used as a rib to improve the bending performance of the bottom plate. It is mainly used in combination with prestressed webs. In this paper, the flange truss composite ribbed plate is adopted [16,17]. The addition of the flange plate can improve the cross-sectional properties of the bottom plate and effectively increase the bending performance before the cracking of the bottom plate.

Currently, the choice of pouring materials is mostly recycled concrete and fiber-reinforced concrete [18,19]. Using recycled concrete pouring, the concrete strength is low, and the bottom plate can hardly meet the design requirements without prestressing [20]. It is necessary to increase the thickness of the web to improve the bending performance of the bottom plate and increase the production cost [21,22]. The research on incorporating fibers in fiber-reinforced concrete includes steel and carbon fibers [23,24,25,26]. The steel fiber-reinforced concrete has the advantages of improving concrete strength, reducing concrete cracks, and inhibiting crack extension, which can significantly improve the bending performance of the bottom plate. Previous scholars have applied it to slab members [18,19]. Studies have shown that steel fiber-reinforced concrete pouring can improve the bending performance, fatigue performance, fire resistance, and punching shear resistance of concrete slabs [27,28,29]. In this paper, high-strength steel fiber-reinforced concrete is used to improve the bending performance of the bottom plate. The amount of steel fiber-reinforced concrete is reduced as much as possible, and the goal of lowering support or even free support during construction is achieved.

For the problem of the high support cost of traditional composite plate construction, the current solution is to apply prestress to the bottom plate so as to reduce support or even free support during construction [30,31]. Many scholars conducted the crack resistance check of the precast floor in the construction stage of the prestressed concrete ribbed composite slab without support design-control conditions and put forward the support concept of this kind of floor in a large-span construction [32,33]. The research results show that the contribution value of prestressed steel bars to the bending performance of prestressed concrete ribbed composite slab floor is greater than that of different forms of ribbed slabs. However, applying prestress to the floor will increase the production cost and make the inverted arch challenging to control. It will also close the cracking load to the failure load, which can easily cause safety problems [31].

**Figure 3:**
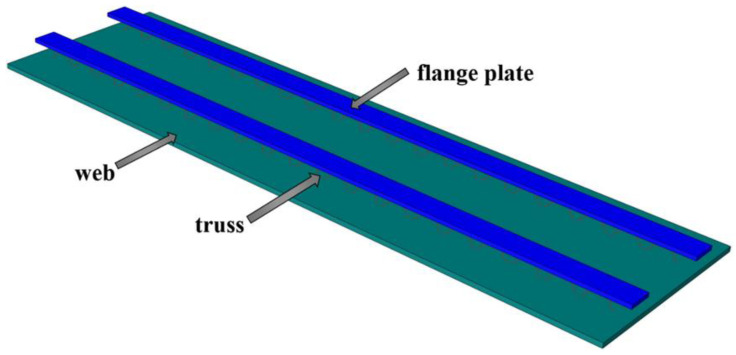
Foundation structure of flange truss composite ribbed slab high-ductility concrete composite slab floor.

For this reason, the flange plate can improve the short-term stiffness of the composite plate bottom plate; steel fiber-reinforced concrete can enhance the strength and flexibility of the floor and increase the crack resistance of the floor. A method of pouring high-ductile concrete (steel fiber volume content of 1%) and using a flange truss composite ribbed plate for a non-prestressed concrete composite slab floor is proposed to achieve the purpose of reducing free support during construction. The floor foundation structure is shown in Figure 3. For the construction of a composite slab without support, the requirement is that the deflection change is less than the calculated span *l*_0_/200, and the bottom plate does not produce cracks when bearing the self-weight load and construction live load of post-pouring layer concrete [34]. When C30 concrete is used for the post-poured layer concrete of the test specimen size, the self-weight load is 2.5 kN/m^2^, and the construction live load is 1.5 kN/m^2^. The bending performance of the CRHDCS under different structures is explored through the test and numerical analysis of four different rib plate structure floors.

## 2. Test Overview

### 2.1. Material Parameters and Overview of the Specimen

#### 2.1.1. Material Properties of Specimen

**Figure 4:**
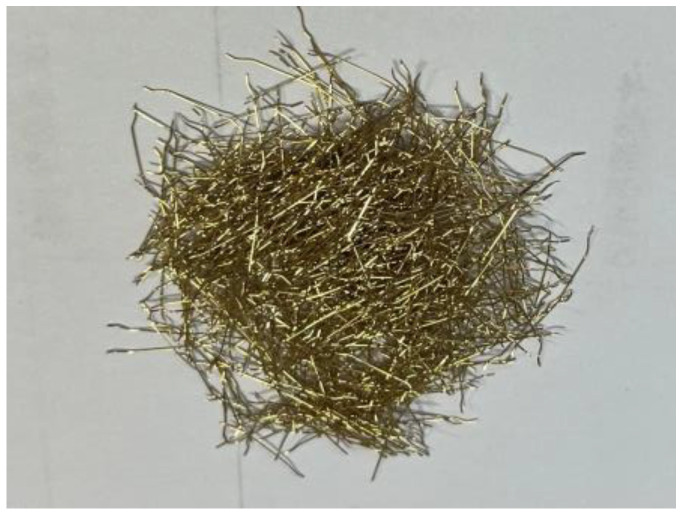
Photograph of steel fiber.

Four full-scale members were made in the test. The pouring materials, steel bars, web sizes, and flange sizes of the four composite slab bottom plates are the same. The pouring material of the composite slab floor is a kind of high-ductility concrete. The concrete is made of cement, fly ash, quartz sand, a water-reducing agent, water, and steel fiber (1% of the volume content) according to the specific ratio, and its designed compressive strength is C60. The steel fiber used is a hook-shaped copper-plated steel fiber with a length of 17 mm and a diameter of 0.44 mm. The specific picture is shown in Figure 4. The standard-size test blocks required for the mechanical properties test of concrete were poured at the same time. After curing, the mechanical properties test was conducted according to the requirements of the “Standard for Testing Methods of Mechanical and Physical Properties of Concrete” (GB/T50081-2019) [35]. The test results are listed in Table 1. Compared with CF60 steel fiber-reinforced concrete with steel fiber volume content of 1%, the cubic compressive strength, axial compressive strength, axial tensile strength, and elastic modulus of high-ductile concrete used in this paper increased by 15%, 18%, 25%, and 10%, respectively. Based on the mechanical properties of the concrete, the research purpose of free support in the construction of a non-prestressed composite slab floor is realized.

#### 2.1.2. Overview of the Specimen

**Figure 5:**
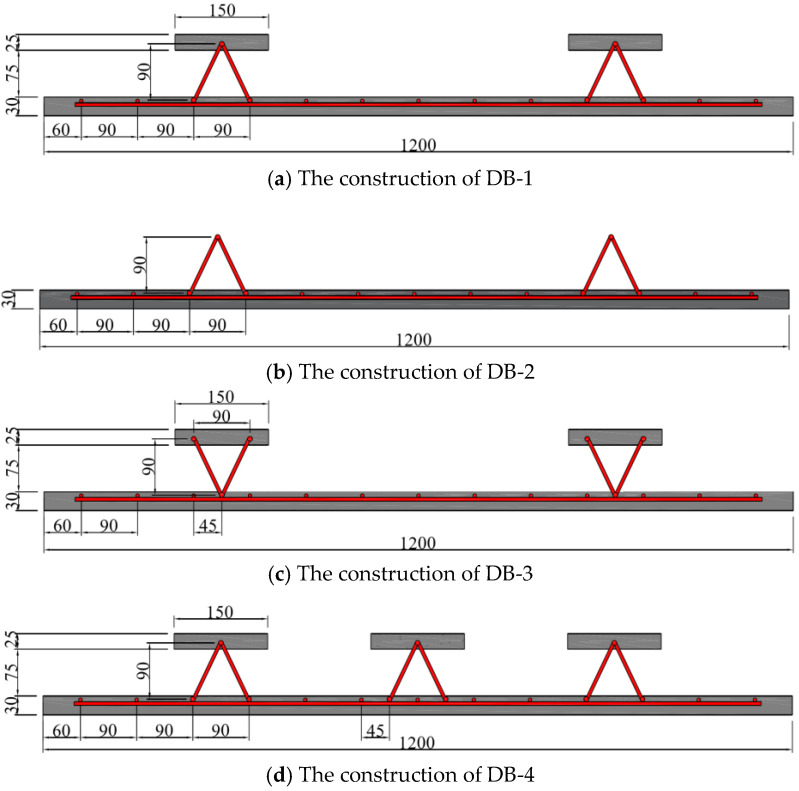
Specimen structure diagram. * The unit of size in the figure is mm.

**Figure 6:**
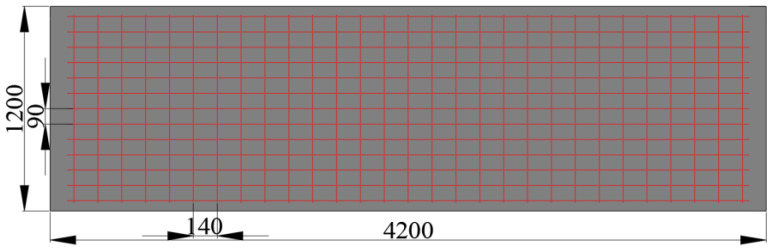
Web structure and size diagram.

The web size of the four test bottom plates is 4.2 m × 1.2 m. The main difference is the rib plate structure of the bottom plate and the influence of different rib plate structures on the bending performance of the bottom plate designed in this paper. The web tensile steel bar and the structural steel bar are configured with the standard load combination during the use period after superposition. The details of each part of the steel bar configuration are shown in Table 2. The abdominal ribs are constructed as DB-1, and three contrast terms are established, which are defined as DB-2, DB-3, and DB-4, respectively. The schematic diagram of each plate structure is shown in Figure 5. The web structure and size diagram are shown in Figure 6. The difference between DB-2 and DB-1 rib plates is that the flange plate is canceled; the difference between DB-3 and DB-1 lies in the inverted truss binding; and DB-4 changed two ribs to three ribs on the rib structure of DB-1. The three comparison items explore the influence of the flange plate, truss-binding method, and number of ribs on the bending performance of the bottom plate of ribbed high-ductile concrete composite plate. The structural schematic diagram of each plate specimen is shown in Figure 2.

### 2.2. Test Scheme

**Figure 7:**
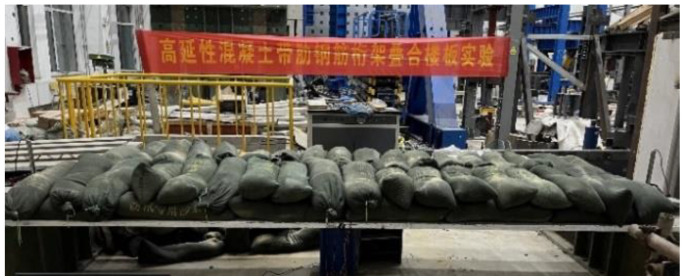
Schematic diagram of the test site.

**Figure 8:**
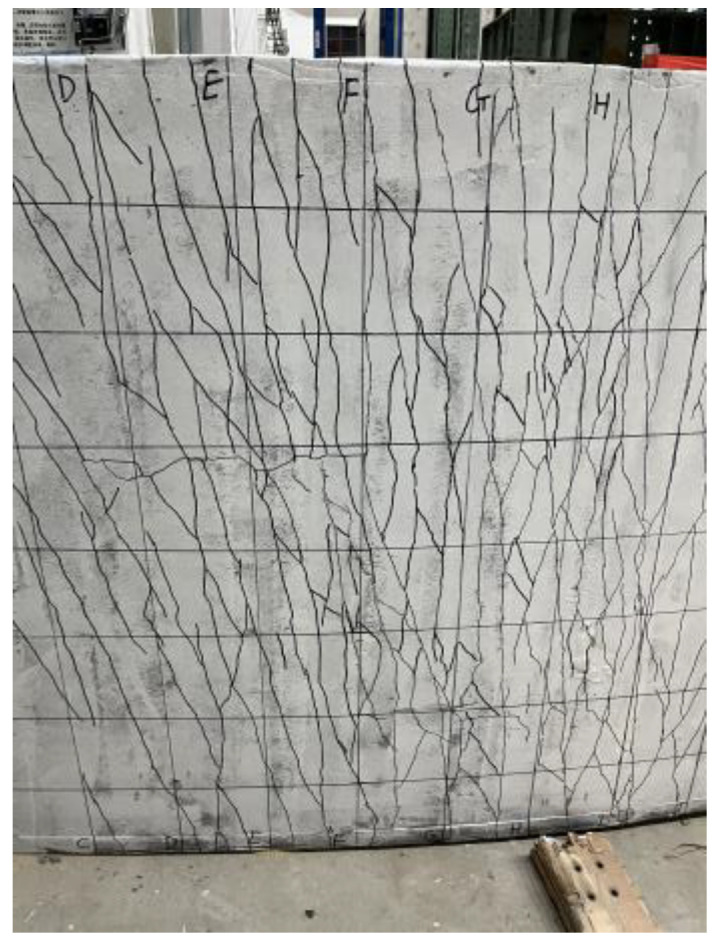
The actual drawing of the test.

The bending performance test of the four kinds of bottom plates is conducted according to the bending performance test requirements of concrete members in the “Concrete Structure Test Method Standard” (GB/T50152-2012) [36]: The simply supported supporting device at both ends is used to test the bending performance of each bottom plate by means of grading and uniform loading. The schematic diagram of the test site is shown in Figure 7. Before the test, the surface of the web was painted with white paint, and a grid with a side length of 150 mm was drawn to mark the cracks during and after the test. The actual drawing of the test is shown in Figure 8. Before the test, the cracking load *q*_cr_ and the ultimate bearing capacity *M*_s_ should be calculated according to the calculation method specified in the “Code for Design of Concrete Structures” (GB50010-2010) [34]. The uniform load was simulated by evenly stacking 20 kg sandbags per bag. In order to prevent the size of ordinary sandbags from being too large, the sandbags are exposed to the edge of the plate during loading, resulting in load concentration. Custom sandbags were used in the test so that the width of the sandbag was equal to the distance between the flange plate edge and the long side of the web when the sandbag was filled with sand.

A preloading test is conducted before formal loading to ensure the stability of the bearing and the regular operation of the test instrument. During the formal loading, 20% of the cracking load *q*_cr_ is used for grading loading. When the loading load reaches 80% *q*_cr_, the load corresponding to the ultimate bearing capacity is 5% *q*_s_ for further loading. After the specimen is cracked, the load is loaded at 10% *q*_s_, and the load is loaded at 5% *q*_s_ when the load is close to *q*_s_. Loading must be stopped in one of the following three cases:

(1) The deflection of the mid-span position of the component reaches 1/50 of the span;

(2) The crack width at the tensile main bar reaches 1.50 mm, or the steel bar strain exceeds 0.01;

(3) Concrete in the compression zone is cracked and broken.

After each stage of loading, the loading time of the specimen is ensured to be longer than 15 min. After the specimen has been cracked, the cracks in the bottom plate are described, and the bearing value is marked at the end of the crack development. The marked bearing value depends on the time of crack occurrence, which can be divided into three certain situations: Cracks appear during the increase of load, during loading, and at the end of loading. For the three cases, the values are taken respectively: the pre-stage load value of this stage loading, the average value of this stage loading and the pre-stage load value, and the load value of this stage.

### 2.3. Layout of Measuring Points

The deflection of the floor and the strain of steel and concrete can be observed in this test. A displacement meter observed the deflection change of the floor. A total of three displacement meters were arranged, which were located at the support ends and mid-spans on both sides. Among them, the displacement gauges at both ends are arranged perpendicular to the upper surface of the web, and the mid-span displacement gauges are arranged perpendicularly to the lower surface of the support.

**Figure 9:**
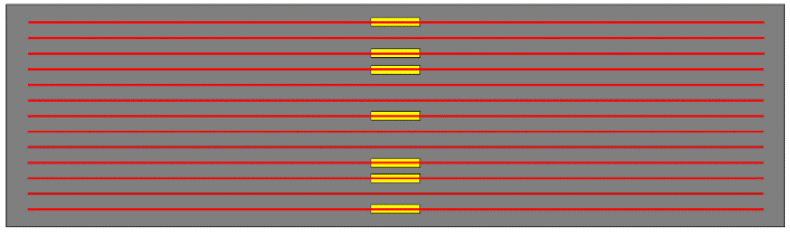
Layout diagram of steel strain gauge.

The strain of steel bars and concrete was measured by the strain gauge. The steel bar strain gauges are pasted before the steel mesh is fixed to the pouring template, which is located on both sides of the tensile steel bar and the central steel bar, the upper and lower chords of the truss span, and the truss web. The specific arrangement points of the tensile steel bar and the upper and lower chord steel bar strain gauges of the steel bar truss are shown in Figure 9.

**Figure 10:**
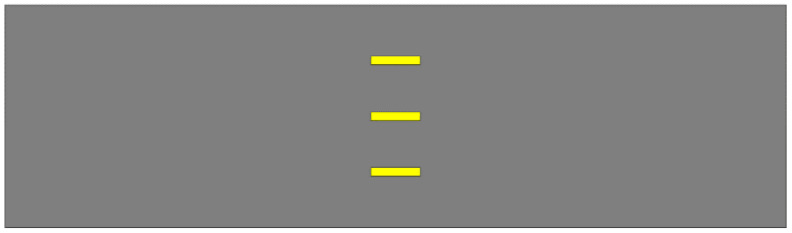
Layout of strain gauges on the lower surface of concrete.

**Figure 11:**
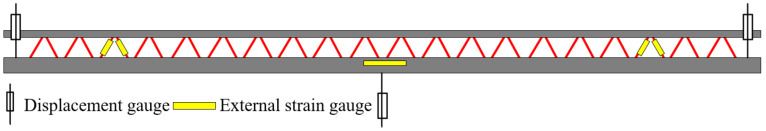
Layout of measuring points on the side surface of concrete.

Three concrete strain gauges are arranged on the middle and lower surfaces of the span. One is located at the center point of the lower surface, and the other two strain gauges are uniformly stuck on its both sides. The distance between the strain gauge and the center strain gauge is 300 mm, and the arrangement points are shown in Figure 10. The measuring points were arranged on the mid-span surface of the concrete to observe the concrete strain on the side surface. The side surface of the web and the arrangement point of the displacement meter are shown in Figure 11.

## 3. Test Results and Analysis

### 3.1. Failure Characteristics and Fracture Distribution

#### 3.1.1. Failure Characteristics of Specimens

Four specimens were conducted to meet the three failure characteristics proposed in this paper 1.2 stop loading, but the failure characteristics of each specimen are different. Among them, the mid-span deflection of specimens DB-1 and DB-4 reached *l*_0_/50 during the application of higher loads, but the tensile steel bars did not yield. At this time, the stop loading was considered as the failure of the specimen, and the ultimate bearing capacity should be taken as the corresponding value of the previous load. The ultimate bearing capacity of the DB-2 specimen should be taken as the corresponding value of the average value of the last load and the current level load because the yield of the tensile steel bar during the current level load is maintained to meet the failure characteristics. Specimen DB-3 is the failure characteristic of the tensile steel bar during the application of the lower load, and the ultimate bearing capacity should be taken as the corresponding value of the previous load.

#### 3.1.2. Crack Distribution of Specimens

**Figure 12:**
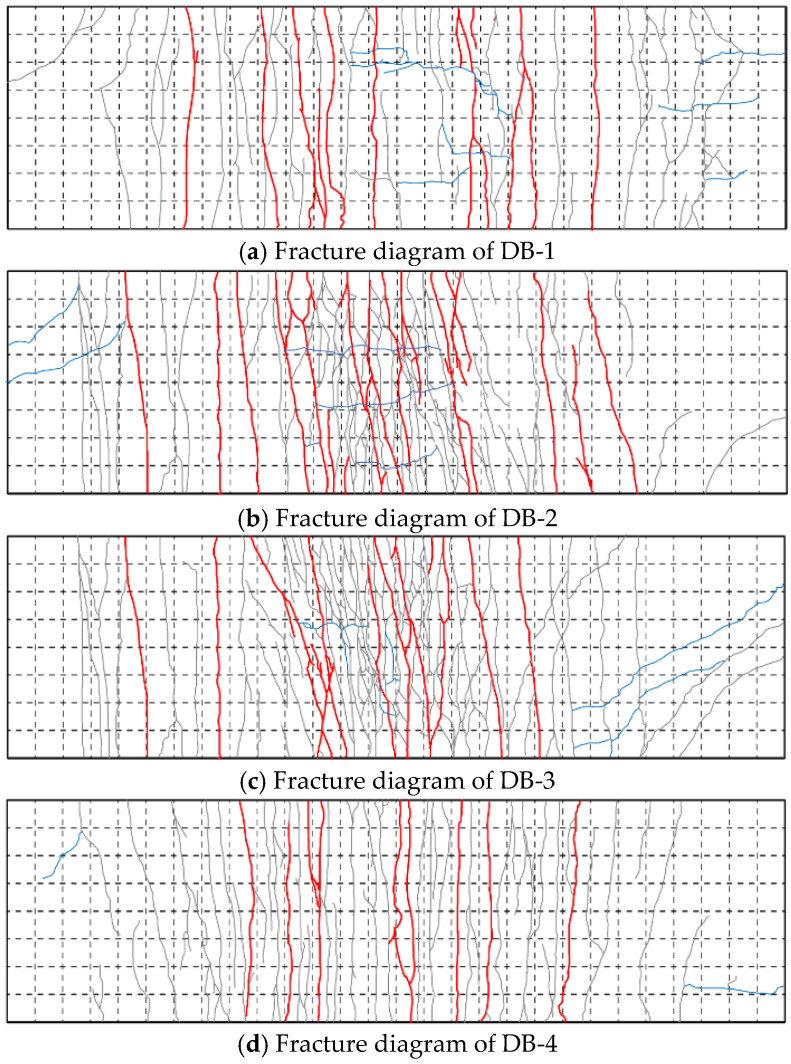
Fracture diagram of the test bottom plate.

The crack labeling work of the four specimens was described, and the width was measured at the end of the loading time. During the test, a crisp sound is generated when new cracks are generated, and the friction sound is caused by the slip of steel fiber and concrete mixed. Because the test will produce minor cracks and cannot be recorded on the whole surface, the segmentation record is conducted according to the grid, and the electronic drawing is conducted in the later stage. Figure 12 shows the electronic drawing of the cracks of each specimen. Most of the cracks in the four specimens are composed of vertical penetrating cracks, “y”-type cracks, and transverse cracks, and the cracks contain steel fibers connecting the concrete on both sides and slipping from the concrete. The concrete steel fiber on both sides of the connection crack is stretched due to the bonding force with the concrete, and the end of the slipped steel fiber is curved.

The first vertical crack of the specimen DB-1 was generated at the approximate mid-span position. A small number of cracks with a width greater than 1 mm were generated in the overall test, and the maximum width of the crack was 1.8 mm. The transverse cracks in the middle span are mainly distributed on both sides of the lower chord steel bar of the truss and are conducted at the end of the “y”-type crack. The crack propagation path near the support is arc-shaped, accompanied by a small amount of small transverse cracks. A large number of vertical cracks with a width greater than 1 mm develop from the web side to the top of the web, and a small number of cracks develop to the top of the plate. The cracks run through the bottom of the flange plate, and the width of the cracks is less than 1 mm. The most extended length of the cracks on the plate side is 5 mm.

The maximum vertical crack width of specimen DB-2 is 2.2 mm. Compared with specimen DB-1, more vertical cracks and “y”-type cracks are generated, and the “y”-type cracks converge in a large number at the mid-span position and extend to the other side of the plate. The DB-2 flange plate of the specimen produces a large number of cracks, including five side-through cracks. The maximum vertical crack width of specimen DB-3 is 2.0 mm. More vertical cracks, “y”-type cracks, and cracks at the proper support are generated than the specimen DB-1. However, there is no arc crack at the left support of the DB-3 specimen. This phenomenon should be caused by the problem of sand leakage in the sandbag used for loading, which makes the actual loading not uniform. The crack development at the flange plate is roughly the same as that of the specimen DB-1, but two penetrating cracks are developed on the plate side. The vertical crack amount of specimen DB-4 is more significant than that of specimen DB-1, but the crack amount of larger width is smaller than that of specimen DB-1, and the maximum crack width is 1.6 mm. Vertical penetrating cracks are generated at the bottom of the flange plates on both sides, and no penetrating cracks are conducted on the sides of the three flange plates. The most extended length of the cracks on the side of the plate is 7 mm.

### 3.2. Analysis of Bending Properties

#### 3.2.1. Analysis of Applied Load and Mid-Span Deflection

The four specimens conform to the elastic stage characteristics of reinforced concrete flexural members before the first crack is generated. As the applied load increases, the growth rate of mid-span deflection is roughly the same, and the applied load-mid-span deflection curve increases according to the approximate slope. As the crack occurs, the specimen enters the elastic–plastic stage. Different from the characteristics of the gradual decrease of the slope of the applied load-mid-span deflection curve in the elastic–plastic stage of conventional flexural members, this paper designs the specimen to enter this stage, and the slope of the applied load-mid-span deflection curve contains one or more slope increase stages. The reason for the analysis is that, in addition to the floor structure form factor, the high-ductile concrete used for pouring the specimen is the main factor causing this phenomenon.

With the continuous increase of the applied load, the cracked cracks continue to extend and develop with the new cracks. With the rise in the number and width of cracks, the tensile load of the disorderly steel fibers distributed in the concrete around the cracks increases suddenly, which makes the steel fibers enter the tensile state. A large number of steel fibers produce tensile stress on the concrete around the cracks, which increases the short-term stiffness of the entire floor structure and reduces the mid-span deflection growth. When the applied load continues to grow, the steel fiber in the crack gap is pulled off or slipped out of the concrete, which reduces the short-term stiffness of the bottom plate and the slope of the load-mid-span deflection curve. As the external load continues to increase, the specimen reaches the ultimate bearing capacity due to the change of mid-span deflection or the yield of the tensile steel bar, which is regarded as a failure.

**Figure 13:**
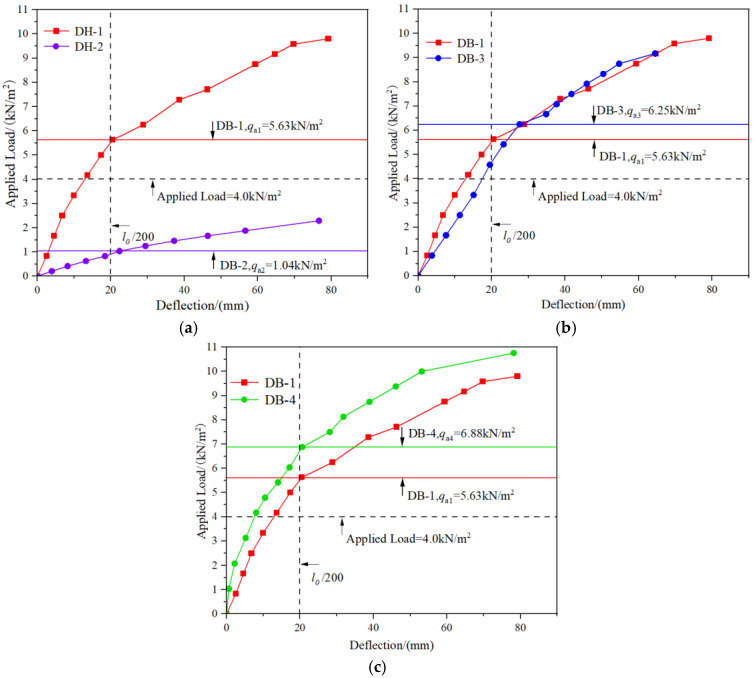
Load-mid-span deflection curve diagram. (**a**) The curves of DB-1 and DB-2; (**b**) The curves of DB-1 and DB-3; (**c**) The curves of DB-1 and DB-4.

In order to facilitate the analysis of the influence of the rib plate structure on the bending performance of the bottom plate, the DB-1-applied load-mid-span deflection curve is drawn in a diagram with the other three specimen curves. According to the curve analysis of the two specimens in Figure 13a, the increase of the flange plate improves significantly the short-term stiffness *B*_0_ and the cracking load *M*_cr_ of the bottom plate. The cracking load *M*_cr1_ = 0.185 *M*_cr2_. DB-1 is not cracked, and the deflection is less than l0/200 when subjected to the self-weight load and live construction load of the composite layer, which meets the requirements of non-support construction. DB-2 has been damaged when bearing the load required by the free support, which does not meet the requirements of the free-support construction. According to the analysis of Figure 13b, the cracking load of the truss with the “V” type is higher than that of the inverted “V” type, but the short-term stiffness is reduced. The low short-term stiffness of DB-3 prevents it from cracking when subjected to the required load, but the deflection is closer to *l*_0_/200. During the construction, the relationship between the deflection, cracking load, and ultimate load of the prefabricated floor should also be considered. According to the analysis of the test results, DB-1’s inverted “V” truss-binding method makes it easier to meet the requirements during construction. According to the curve shown in Figure 13c, the increase in the number of ribs improves the bending performance of DB-4 compared with DB-1, and the cracking load *M*_cr4_ = 1.22 *M*_cr1_. In this test, DB-1 is more suitable for the performance application of DB-4 under external load. If it is necessary to bear the more significant external load, it can be selected to increase the number of ribs to meet the construction requirements.

#### 3.2.2. Analysis of Applied Load and Mid-Span Strain

**Figure 14:**
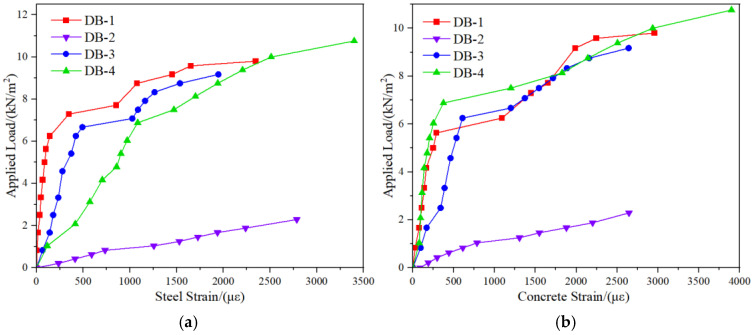
Load-strain curve at mid-span. (**a**) Load-web mid-span steel strain curve; (**b**) External load-web mid-span concrete strain curve.

Figure 14a shows the applied load–strain curve of the mid-span steel bar. The strain of the steel bars in the mid-span of the four specimens maintained an approximately stable growth rate before reaching the cracking moment. As the load continued to increase after cracking, the bottom plate and the shaft moved up, the short-term stiffness of the structure decreased, and the strain-increase rate gradually increased. The slope of the continuous loading curve after cracking of the designed bottom plate in this paper is not a fixed reduction phenomenon, and the influence of steel fiber on the stiffness of the bottom plate is analyzed. When the load increases to a specific value, a large number of steel fibers are pulled off, and the tensile steel bar bears most of the tensile stress so that the strain-growth rate of the web tensile steel bar continues to increase. By comparing DB-1 and DB-3 curves, it can be analyzed that the “V”-type binding of truss steel bars reduces the cross-sectional area of tensile steel bars in the bottom plate and increases the cross-sectional area of compressive steel bars. According to the test results, this structural change will reduce the short-term stiffness of the bottom plate and increase the potential safety hazard of free support during construction. The load-deflection curve analysis shows that the short-term stiffness of the bottom plate is effectively improved by expanding the rib plate. For this reason, the steel bar strain-change curve does not conform to the results of the previous analysis. The reason should be that when the sandbag is loaded for the second time because the flange plate spacing is less than the sandbag width, the sandbag is directly placed on the top of the flange plate to form a concentrated force transmitted to the web tensile steel bar so that the strain suddenly increases. This problem will not occur during the pouring of the laminated layer, which can be further defined according to the previous conclusions.

The variation law of the applied load-tension zone mid-span concrete strain curve is approximate to that of the steel bar curve. The curve is a constant slope before the cracking of the bottom plate and gradually decreases after cracking. Comparing the curve provided in Figure 14b, the applied load-tension zone mid-span concrete strain curve of DB-1 and DB-4 can further prove the previous analysis of the variation law of the applied load-web mid-span steel bar-strain curve: the increase in the number of flange plates can increase the cracking moment and short-term stiffness of the bottom plate. Combined with the mid-span steel and concrete strain curves of each bottom plate, only the two curves of DB-4 always keep decreasing and developing after the bottom plate cracks. This linear change is the same as that of conventional concrete flexural members, indicating that the rib plate-reconstruction method of increasing the number of rib plates will reduce the utilization rate of high-ductile concrete characteristics after the crack of the rib plate.

## 4. Numerical Analysis

**Figure 15:**
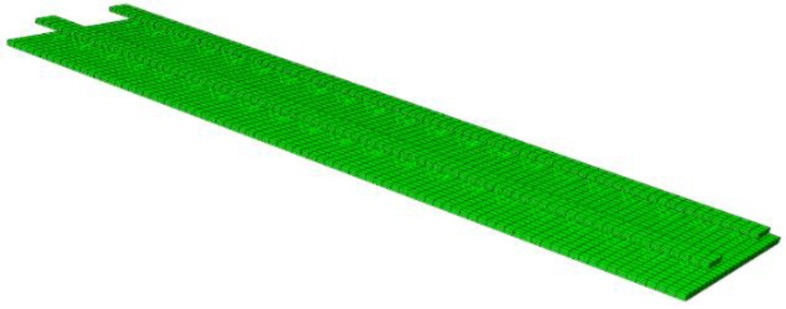
Numerical model diagram (DB-1).

Based on the theory of the numerical element method, finite-element analysis software is used to simulate and analyze the composite floor with four different rib structures designed in this paper. By comparing them with the laboratory test data, the feasibility and rationality of the test results for the bending performance of the flange truss high-ductility concrete composite slab floor are verified. The geometric model of the laminated floor is divided into the following parts: web, flange plate, web longitudinal tensile steel bar and distributed steel bar, truss steel bar, and bearing block. According to the actual layout, the assembly is conducted to form the numerical model of the bottom plate (Figure 15 is the DB-1 numerical model). The grid size of the concrete part of the precast floor is 60 × 50 mm. The grid size of the steel bar in the prefabricated bottom plate is 50 mm. During the test, the bearing of the specimen is simply supported. Therefore, when defining the boundary conditions, the load and constraint are applied to the reference point of the discrete rigid body.

The concrete adopts the plastic damage model provided by ABAQUS, and the elastic–plastic constitutive of steel bars adopts the isotropic elastic–plastic model provided by ABAQUS. According to the constitutive relationship of concrete in Reference 35, the uniaxial stress–strain relationship is determined; that is, Equations (1) to (4). The “plastic” parameter value is as shown in Table 3.

When pulled:

(1) $$\sigma =(1-d_{t})E_{c}\varepsilon$$

(2) $$d_{t}=\left\{\begin{array}{l} 1-\rho_{t}\left[1.2-0.2x^{5}\right],\;x\leq 1\\ 1-\frac{\rho_{t}}{\alpha_{t}{\left(x-1\right)}^{1.7}+x},\;x>1 \end{array}\right.$$

where $x=\frac{\varepsilon }{\varepsilon_{t,r}}$, $\rho_{t}=\frac{f_{t,r}}{E_{c}\varepsilon_{t,r}}$. *E*_c_ is the elastic modulus of concrete; *α*_t_ is the parameter of the descending section of uniaxial tensile stress–strain curve of concrete numerical value; *f*_t,r_ is the uniaxial tensile strength of concrete; *ε*_t,r_ is the peak tensile strain of uniaxial tensile strength of concrete; *d*_t_ is uniaxial tensile damage evolution parameters of concrete.

When under pressure:

(3) $$\sigma =(1-d_{c})E_{c}\varepsilon$$

(4) $$d_{c}=\left\{\begin{array}{l} 1-\frac{\rho_{c}n}{n-1+x^{n}},\;x\leq 1\\ 1-\frac{\rho_{c}}{\alpha_{c}{\left(x-1\right)}^{2}+x},\;x>1 \end{array}\right.$$

where $\rho_{c}=\frac{f_{c,r}}{E_{c}\varepsilon_{c,r}}$, $n=\frac{E_{c}\varepsilon_{c,r}}{E_{c}\varepsilon_{c,r}-f_{c,r}}$, $x=\frac{\varepsilon }{\varepsilon_{\;c,r}}$. *α*_c_ is the parameter of the descending section of the uniaxial compressive stress–strain curve of concrete numerical value; *f*_c,r_ is the uniaxial compressive strength of concrete; *ε*_c,r_ is the peak tensile strain of uniaxial compressive strength of concrete; *d*_c_ is damage evolution parameters of concrete under uniaxial compression.

The stress–strain curve of the steel bar under monotonic loading is determined according to Formula (5).

(5) $$\sigma_{s}=\left\{\begin{array}{l} E_{s}\varepsilon_{s},\;\varepsilon_{s}\leq \varepsilon_{y}\\ f_{y,r}+k(\varepsilon_{s}-\varepsilon_{y}),\;\varepsilon_{y}<\varepsilon_{s}\leq \varepsilon_{u}\\ 0\;,\varepsilon_{s}>\varepsilon_{u} \end{array}\right.$$

where *E*_s_ is the elastic modulus of steel bar; σ_s_ is the stress of steel bar; *f*_y,r_ is the yield strength of steel bar; *f*_st,r_ is the ultimate strength of the steel bar; ε_y_ is the yield strain of the steel bar; ε_uy_ is the strain of the steel bar hardening point; ε_u_ is the ultimate strain of the steel bar; and *k* is the slope of the hardening section.

Through the numerical simulation analysis of the bending performance of the four bottom plates, the cracking load *q*_a_ of each plate is compared with the test cracking load *q*_t_ (Table 4). The analysis results show that the maximum error between the numerical simulation cracking load of each floor and the test results is 10%.

**Figure 16:**
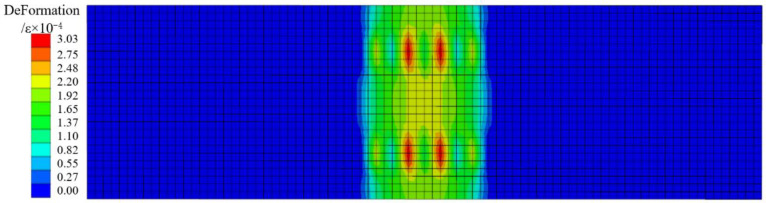
Plastic strain program of concrete (DB-1).

**Figure 17:**
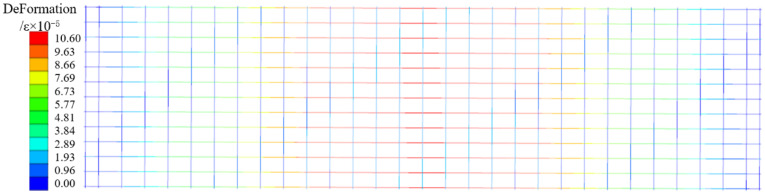
Steel strain cloud diagram (DB-1).

Figure 16 and Figure 17 are the concrete, plastic strain cloud map, and the steel bar strain cloud map of DB-1 under the numerical simulation cracking load. The main strain distribution is the same as the test results, and the strain is concentrated in the mid-span part. The maximum strain of concrete is concentrated near the bottom chord of the truss, the ratio of the mid-span strain to the test result is 1.04, and the ratio of the mid-span strain of the steel bar to the test result is 1.05. After comparing the simulation results of the four specimens with the experimental results, it is found that the error of cracking load and short-term stiffness obtained by the four-plate simulation is less than 10%, and both are smaller than the experimental results, indicating that the database used for this numerical model cannot perfectly reflect the bending performance of steel fiber to the bottom plate under actual conditions. For this error, I think the possible reason is that the ordinary concrete constitutive model cannot simulate the influence of steel fiber on the bending performance of the bottom plate in the actual test process. The steel fiber model can be established separately to distribute it irregularly in the concrete model of the bottom plate. In the process of the test simulation, it cooperates with concrete to reduce the error with the test data. The bending performance analysis results of the above numerical simulation are compared with the experimental results, and the back bending error is small. It indicates that the experimental analysis results of the bending performance of the flange truss high-ductility concrete composite slab floor are reliable.

## 5. Non-Support Calculation Method

### 5.1. Derivation of Related Parameters of Support-Free Theory

**Figure 18:**
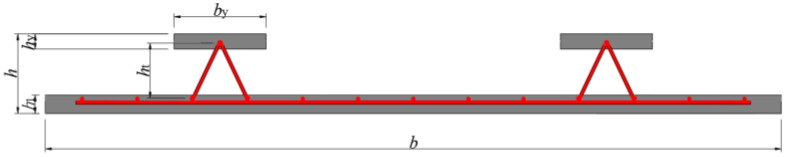
Flange rib high-ductile concrete composite slab floor stiffness calculation diagram.

By comparing the laboratory test results with the numerical simulation results, it can be proved that the test data are reliable. According to the test results, the main influencing factors are the cracking moment *M*_cr_ of the bottom plate and the short-term stiffness *B*_0_ before cracking. Firstly, the neutral axis position should be calculated according to the section properties. The calculation diagram is shown in Figure 18. *x* is set to the distance from the neutral axis to the edge of the tensile zone. For similar types of cross sections, *x* can be calculated according to Equation (6).

(6) $$x=\frac{\frac{b}{2}h_{1}^{2}+(a_{E}-1)A_{s}(x-c_{1})+(a_{Et}-1)A_{\mathrm{sd}}(x-c_{2})+(a_{Et}-z)A_{s}\prime (h_{t}+c_{2}-x)+nb_{y}h_{y}(h-\frac{h_{y}}{2})}{bh_{1}+(a_{E}-1)A_{s}+(a_{Et}-1)A_{\mathrm{sd}}+(a_{Et}-z)A_{s}\prime +nb_{y}h_{y}}$$

where *b* is the width of the web; *h*_1_ is the web thickness; *c*_1_ and *c*_2_ are the centroid of the longitudinal steel bar in the web and the distance from the bottom chord of the truss to the edge of the tension zone; *h*_t_ is the height of the truss; *b*_y_ is the width of the flange plate; *h*_y_ is the thickness of the flange plate; *h* is the total thickness of the floor; *a*_E_ is the elastic modulus ratio of web longitudinal reinforcement to concrete; *a*_Et_ is the elastic modulus ratio of the upper and lower chords of the truss to the concrete; and *A*_s_, *A*_sd,_ and *A*_s_′ are the cross-sectional area of tensile steel bar, truss lower chord steel bar, and compressive steel bar. *n* is the number of flange plates; *z* is the control coefficient of the cross-sectional area of the compressive steel bar. If *n* is equal to 0, then *z* is equal to 0. If *n* is greater than 0, then *z* is equal to 1.

The moment of inertia of section *I*_0_ is the sum of the moment of inertia of the two sections of the concrete and the steel bar to the neutral axis; that is, Equation (7):

(7) $$I_{0}=I_{0c}+I_{0s}$$

where *I*_0c_ and *I*_0s_ are the moments of inertia of the concrete section and the steel bar section to the neutral axis, respectively; the calculation formulas are shown in Equation (7a) and Equation (7b), respectively:

(7a) $$I_{0c}=\frac{b}{3}\left[x^{3}-{\left(x-h_{1}\right)}^{3}\right]+\frac{nb_{y}}{3}\left[{\left(h-x\right)}^{3}-{\left(h-x-h_{y}\right)}^{3}\right]$$

(7b) $$\begin{array}{l} I_{0s}=(a_{E}-1)A_{s}{\left(x-c_{1}\right)}^{2}+(a_{Et}-1)A_{s}{\left(x-c_{2}\right)}^{2}\\ \;\;+(a_{Et}-z)A_{s}^{\prime }{\left(h_{t}+c_{2}-x\right)}^{2} \end{array}$$

If the building floor is made of conventional concrete, the cracking moment *M*_cr_ and the short-term stiffness *B*_0_ before cracking can be calculated according to the formula specified in the “code for design of concrete structures” (GB50010-2010) [34]. The specific formula is shown in Equations (8) and (9).

(8) $$M_{\mathrm{cr}}=\gamma f_{\mathrm{tk}}W_{0}$$

(9) $$B_{0}=0.85E_{c}I_{0}$$

where *γ* is the plastic influence coefficient of the section resistance moment of the concrete member; *f*_tk_ is the standard value of axial tensile strength of concrete; and *W*_0_ is the elastic resistance moment of the tensile edge of the converted section of the member, which is the quotient of the moment of inertia of the converted section and the distance from the neutral axis to the edge of the tensile zone; that is, *I*_0_/*x*. *E*_c_ is the elastic modulus of concrete.

The values of *M*_cr_ and *B*_0_, calculated according to Equations (8) and (9), have significant errors in the experimental data, indicating that they are not suitable for the flange truss high-ductility concrete composite slab floor. Through the above formula, it can be seen that *M*_cr_ and *B*_0_ are related to section properties and material properties. It is speculated that the main factor is *M*_cr_. According to the “Technical Specification for Fiber Reinforced Concrete Structures” (CECS 38-2004) [37], the axial tensile standard value *f*_ftk_ of steel fiber-reinforced concrete should be converted according to Equation (10):

(10) $$f_{\mathrm{ftk}}=f_{\mathrm{tk}}(1+\alpha_{t}\lambda_{t})$$

where *α*_t_ is the influence coefficient of steel fiber on the axial tensile strength of steel fiber-reinforced concrete; *λ*_t_ is the characteristic value of steel fiber content, which is determined by the parameters of steel fiber materials. The specific calculation formula is Equation (10a):

(10a) $$\lambda_{f}=\rho_{f}l_{f}/d_{f}$$

where *ρ*_f_ is the volume fraction of steel fiber; lf is the length of steel fiber; *d*_f_ is the diameter of steel fiber.

The corrected cracking moment *M*_fcr_ is calculated according to Equation (11):

(11) $$M_{\mathrm{fcr}}=\gamma f_{\mathrm{ftk}}W_{0}$$

According to the test results, the *B*_0_ error factor is the influence of material properties and truss web members on the short-term stiffness of the bottom plate. For the impact of the truss web member on the short-term stiffness of the bottom plate, Y. L. Liu [38] proposed an equivalent idea. The spatial three-dimensional truss steel bar was simplified into a plane truss model by using the substructure method. On this web, the equivalent stiffness principle was applied to derive the contribution of the inverted “V” truss web member to the bending stiffness of the specimen. The equivalent bending stiffness *B*_st_ of the inverted “V” truss single web member to the specimen is:

(12) $$B_{\mathrm{st}}=\frac{11E_{t}A_{t}a^{2}\sin2\theta }{24}$$

where *E*_t_ is the elastic modulus of the truss web member; *A*_t_ is the cross-sectional area of the web member; *a* is 50% of the span of the single-span truss; and *θ* is the angle between the web member and the upper and lower chord steel bars in the equivalent model diagram.

**Figure 19:**
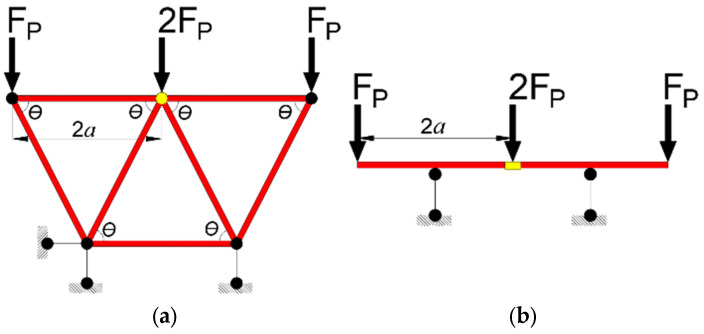
Truss equivalent calculation diagram. (**a**) Simplified truss model; (**b**) Equivalent beam model diagram.

According to the principle that the displacement of point A in the simplified model (Figure 19a) and the equivalent beam model (Figure 19b) is equal (the yellow mark in the diagram is point A), the calculation formula of the equivalent bending stiffness *B*_st_′ of the single web member of the “V” truss to the specimen is obtained, as shown in Equation (13):

(13a) $$\frac{F_{P}a}{E_{t}A_{t}\sin^{2}\theta \cos\theta }=-\frac{F_{P}a^{3}}{6E_{b}I_{b}}$$

Therefore, the formula for calculating the equivalent bending stiffness *B*_st_′ of a single web member to the specimen is:

(13b) $$B_{\mathrm{st}}\prime =E_{b}I_{b}=-\frac{E_{t}A_{t}a^{2}\sin2\theta }{12}$$

where *E*_b_ is the equivalent elastic modulus of the beam; *I*_b_ is the inertia moment of the equivalent beam section.

After comprehensively considering the influence of material properties and truss web members on the short-term stiffness of the bottom plate, the short-term stiffness *B*_s_ formula of the flange truss high-ductile concrete composite plate before cracking is summarized:

(1) If the truss binding method is an inverted “V”-type truss:

(14a) $$B_{s}=B_{0}(1+\beta_{t}\lambda_{t})+nB_{\mathrm{st}}$$

where *β*_t_ is the influence coefficient of steel fiber on the short-term stiffness of reinforced steel fiber-reinforced concrete flexural members.

(2) If the truss binding method is a ‘V’ type truss:

(14b) $$B_{s}=B_{0}(1+\beta_{t}\lambda_{t})+nB_{\mathrm{st}}\prime$$

### 5.2. Method Validation and Analysis

According to the section properties of the specimens designed in this paper and the previous Equations (6) and (7), the position of the neutral axis and the moment of inertia of the converted section are calculated and then substituted into Equations (11) and (14) to calculate the cracking moment and the short-term stiffness before cracking of each specimen after correction, and the results are compared with the test results. Table 5 shows the comparison between the measured cracking moment *M*_t_ and the calculated cracking moment *M*_c_.

The comparison results of the four specimens in Table 4 show that the maximum error between the modified cracking moment calculation Equation (11) and the measured value is 6%, which meets the requirements of engineering calculation accuracy. The influence rate of the cracking moment of the contrast abdominal structure is −81.5%, 11.0%, and 22.2% by removing the flange plate, truss inverted binding, and adding the rib plate.

According to the Equation (14), the short-term stiffness *B*_c_ before cracking of the four plates is calculated, and the measured short-term stiffness *B*_t_ before cracking is calculated according to the Equation (15) combined with the test results. Comparing the two calculation results, the maximum error is 8%, which meets the requirements of engineering calculation accuracy.

(15) $$B_{t}=\frac{5ql_{0}^{4}}{382f}$$

where *q* is the external load; *f* is the mid-span deflection change.

By comparing and analyzing the short-term stiffness results of DB-1, DB-2, DB-3, and DB-4 before cracking calculated by Equation (15), based on the short-term stiffness of DB-1, the influence rate of three structural forms on the short-term stiffness of the floor is analyzed. The lack of a flange plate greatly influences the short-term stiffness of the bottom plate, and the influence rate is 87.6%. The inverted truss binding will slightly reduce the short-term stiffness of the bottom plate, and the influence rate is −1.5%. The short-term stiffness is greatly improved by adding ribs, and the influence rate is 37.5%. Therefore, support-free construction can be realized by rationally designing the flange truss high ductility concrete composite slab floor. Through the influence rate of different structures on cracking bending moment and short-term stiffness before cracking, for the composite slab floor proposed in this paper, the flange truss composite rib should be selected in the design, and the rib should be tied in an inverted ‘V’ shape. When the actual construction is carried out, if the construction live load or the dead weight of the post-pouring layer increases slightly compared with the load combination value specified in this paper, the performance of the bottom plate can be increased by increasing the width or thickness of the flange plate; if the load combination value is much larger than that specified in this paper, the bottom plate design should be carried out by adding a combined rib plate.

According to the test results and the modified formula, the design method of setting the flange plate on the steel bar truss can improve the section properties of the bottom plate. The floor pouring material is a kind of high ductility concrete made of a specific mix ratio. This concrete has higher axial tensile strength and elastic modulus than ordinary steel fiber reinforced concrete. Both of these can improve the cracking load and short-term stiffness of the floor before cracking, so that the floor can meet the requirements of no support during construction.

## 6. Conclusions

In this paper, a flange truss high ductility concrete composite slab floor is proposed. Through laboratory tests, numerical analyses, and related parameter calculations, the bending performance and working performance of the flange truss high ductility concrete composite slab floor under different rib structures are explored. The calculation formula of the critical coefficient before cracking is modified, which provides a reference for the subsequent structural design of the same type of floor for the realization of free support construction. The main conclusions are as follows:

(1) The bending performance of a flange truss high ductility concrete composite slab bottom plate presents two evident stages, with the bottom plate cracking being the turning point. After cracking, due to the use of high-performance concrete and the addition of a flange plate, the short-term stiffness of the floor is different from that of the conventional floor, The short-term stiffness includes the form of periodic increase.

(2) The bending performance comparison of four rib structure forms of flange truss high ductility concrete composite slab floor. The results show that the addition of a flange plate, which is different from that of a prestressed concrete composite slab, has a significant influence on the bending performance of flange truss high ductile concrete composite slab. The ‘V’ type binding of the truss will increase the cracking load of the bottom plate and reduce the short-term stiffness of the bottom plate. Adding more ribs can effectively improve the cracking load and short-term stiffness, but it wastes performance and increases the construction cost in disguise.

(3) The numerical simulation analysis of the bending performance of the four rib structure forms for the CRHDCS is carried out. The analysis results are the same as the experimental ones (the maximum error is 10%). The comparison of simulation and test results shows that the cracking load and short-term stiffness decrease, and the bending performance decreases gradually after cracking. This phenomenon indicates that the conventional model of concrete slab members established by the mechanical properties of high ductile concrete cannot reflect the actual situation of high ductile concrete slab bending, and measures should be taken to solve it.

(4) The formula for the cracking moment and short-term stiffness before the cracking of the no support during construction is modified. Compared with the test results, the maximum error of the modified formula of the cracking moment is 6%. The influence rate of the cracking moment of the contrast abdominal structure is −81.5%, 11.0%, and 22.2% by removing the flange plate, truss inverted binding, and adding the rib plate. The equivalent short-term stiffness formula of the single web member of the ‘V’ truss to the specimen is established. The maximum error of the short-term stiffness correction formula is 8%. The influence rates of the three comparative structural forms on the short-term stiffness of the design standard structural form in this paper are −87.6%, −1.5%, and 37.5%, respectively.

## Figures and Tables

**Table 1:** Test results of mechanical properties of high-ductile concrete.

Mechanical Property	*f*_cc_/(N/mm^2^)	*f*_cp_/(N/mm^2^)	*f*_ts_/(N/mm^2^)	*f*_f_/(N/mm^2^)
measured value	75.70	55.60	5.81	11.38

* *f*_cc_ is the measured cube compressive strength; *f*_cp_ is the measured axial compressive strength; *f*_ts_ is the measured splitting tensile strength; *f*_f_ is the measured flexural strength.

**Table 2:** Details of the bottom plate-reinforcement configuration.

Reinforcement Location	Configuration Details
Web longitudinal reinforcement	A6@90
Web distribution of steel bars	A6@140
Truss upper and lower chord steel bar	C8
Truss web reinforcement	A6

**Table 3:** Plastic parameter value.

Parameter	Expansion Angle	Eccentricity	K Coefficient	Viscosity Parameter
The value	30°	0.1	2/3	0.00045

**Table 4:** Cracking load comparison results of test and numerical simulation.

Test Piece	*q*_t_/kN/m^2^	*q*_a_/kN/m^2^	*q*_a_/*q*_t_
DB-1	5.63	5.25	0.93
DB-2	1.04	1.01	0.97
DB-3	6.25	5.63	0.90
DB-4	6.88	6.27	0.91

**Table 5:** Comparison of measured cracking moment and calculated cracking moment results.

Test Piece	M_t_ (kN/m)	M_c_ (kN/m)	M_t_/M_c_
DB-1	13.51	14.01	0.96
DB-2	2.50	2.53	0.99
DB-3	15.00	14.14	1.06
DB-4	16.51	16.04	1.03

## Data Availability

The original contributions presented in this study are included in the article. Further inquiries can be directed to the corresponding author(s).
